# Effects of Isoacid Supplementation on In Vitro Rumen Fermentation, Nutrient Degradability and Bacterial Community Diversity Using Corn Silage–Highland Barley Straw as Substrates in Yaks

**DOI:** 10.3390/microorganisms14030692

**Published:** 2026-03-19

**Authors:** Can Luo, Fei Jiang, Anyi Zhong, Xinjue He, Xi Liu, Yanling Huang, Yanhua Gao

**Affiliations:** 1 College of Animal Science and Veterinary Medicine, Southwest Minzu University, Chengdu 610041, China; 202330903063@stu.swun.edu.cn (C.L.); 80400015@swun.edu.cn (F.J.); 18759368730@163.com (A.Z.); 202330903057@stu.swun.edu.cn (X.H.); swunylh@163.com (Y.H.); 2 Key Laboratory of Animal Science of National Ethnic Affairs Commission, Southwest Minzu University, Chengdu 610041, China; 3 Key Laboratory of Qinghai-Tibetan Plateau Animal Genetic Resource Reservation and Utilization of Ministry of Education, Southwest Minzu University, Chengdu 610041, China; 4 Institute of Animal Husbandry of Ganzi Tibetan Autonomous Prefecture of Sichuan Province, Kangding 626000, China; ailikey@163.com

**Keywords:** feed additives, isoacids, in vitro gas production, fermentation parameters, microbiota, rumen

## Abstract

This study investigated the effects of isoacid supplementation on in vitro rumen fermentation characteristics, nutrient degradability, and bacterial community diversity in yaks using corn silage–highland barley straw-based substrates. An in vitro fermentation experiment was conducted with a substrate consisting of 80% whole-plant corn silage and 20% highland barley straw. Treatments included a control (without isoacids) and four isoacid supplemental levels (0.1%, 0.2%, 0.3%, and 0.4% of substrate dry matter, DM), each with six replicates. A 72 h in vitro gas production experiment was performed to measure cumulative gas production, fermentation parameters, nutrient degradability, and bacterial community diversity. Cumulative gas production increased by 12.96% with 0.2% isoacid supplementation compared to the control (*p* < 0.05). The contents of microbial protein (MCP), acetate, propionate, and total volatile fatty acids (TVFA) exhibited quadratic responses to the increasing isoacid dosage (*p* < 0.05). Specifically, MCP content reached a maximum of 0.76 mg/mL with 0.2% isoacids, representing a 31.03% increase compared to the control (*p* < 0.05). TVFA content was highest (146.85 mmoL/L) at 0.2% isoacid supplementation, with a 16.40% increase compared to the control (*p* < 0.05). Acetate content increased by 17.99% (*p* < 0.05), while propionate tended to increase with 0.2% isoacid supplementation (*p* = 0.08). Supplementation with 0.2% and 0.4% isoacids did not alter the bacterial composition and diversity (*p* > 0.05). However, at the genus level, g_*Ruminococcus*, g__*Elusimicrobium*, g_norank_f_*Atopobiaceae*, g_norank_o_*Coriobacteriales*, and g_*Romboutsia* were identified as differential biomarkers showing significant responses to isoacid supplementation (*p* < 0.05). Mantel-test analysis revealed positive correlation between g_*Ruminococcus* abundance and NH_3_-N content (*r* < 0.4, *p* < 0.05); g_*Romboutsia* abundance and acetate content (*r* < 0.40, *p* < 0.05); g_*Defluviitaleaceae*_UCG-011 abundance and both NH_3_-N content and the pH of rumen fluid (*r* < 0.40, *p* < 0.05); g_norank_o_*Coriobacteriales* abundance and rumen pH (*r* < 0.40, *p* < 0.01). Supplementation with 0.2% isoacids to corn silage–barley straw substrates improved in vitro rumen fermentation characteristics in yaks, which was associated with altered abundances of key bacterial genera including g_*Ruminococcus*, g__*Elusimicrobium*, g_norank_f*_Atopobiaceae*, g_norank_o_*Coriobacteriales*.

## 1. Introduction

Yaks (*Bos grunniens*), as endemic ruminants of the Qinghai–Tibet Plateau, are highly adapted to alpine and hypoxic environments, providing local residents with essential resources including milk, meat, wool, fuel, and labor [1]. However, seasonal shortages of forage and inadequate feed supply during the long winter season frequently result in insufficient nutrient intake, severely limiting the growth performance and productivity of yaks. Therefore, developing and utilizing local roughage resources is of great social and ecological significance for ensuring healthy production and promoting sustainable development of yak husbandry on the plateau areas.

Highland barley straw, a major crop extensively cultivated in the Qinghai–Tibet Plateau, contributes 43% of the region’s total grain production [2]. Highland barley straw is a potential resource of forage feed for ruminants. However, its high lignocellulose and low protein content severely restrict its nutritional value. Therefore, enhancing the utilization of highland barley straw is crucial for alleviating feed resource shortages in the plateau region [3]. Whole-plant corn silage is globally recognized as premier forage source, which features high carbohydrate content, excellent fermentation characteristics, and high digestibility [4,5]. When combined with highland barley straw for feeding, it can compensate for the straw’s high fiber and low protein content, thereby improving the overall utilization efficiency of the mixed forages. For example, in dairy calves, feeding barley straw in diets before and after weaning was more effective in promoting concentrate feed intake after weaning [6]. Although this mixed-forage strategy has been adopted on some yak farms, its utilization efficiency remains suboptimal. Therefore, enhancing the feed utilization efficiency of mixed roughage through nutritional modulation is essential for advancing yak husbandry in the Qinghai–Tibet Plateau region.

Isoacids, also known as branched-chain volatile fatty acids (BCVFAs), comprise isobutyrate, isovalerate, and 2-methylbutyrate-microbial metabolites derived from valine, leucine, isoleucine, and proline [7,8]. Previous research has demonstrated that supplementing low-fiber diets with 2.15 mmol/d of mixed isoacids promotes MCP synthesis and improves rumen fermentation characteristics [9]. Moreover, isoacid supplementation has improved growth performance and average daily gain (ADG) in dairy cows fed high-fiber diets while increasing the content of odd-chain fatty acids in milk [10]. Furthermore, isoacid supplementation influences feed sorting, chewing behavior, and enteric methane emissions in mid-lactation cows, with effects modulated by dietary fiber levels [7]. In our previous yak study, supplementing a total mixed ration (TMR, with concentrate to roughage ratio at 50:50) with 0.3% mixed isoacids enhanced the synthesis of MCP and volatile fatty acids (VFAs), improved rumen fermentation, and increased growth performance [11]. Collectively, these findings suggest that isoacid efficacy on rumen fermentation is modulated by substrate composition, isoacid type, and supplementation dosage.

Based on this, we hypothesized that supplementing an appropriate dosage of isoacids to corn silage–highland barley straw substrate would enhance in vitro rumen fermentation performance, improve nutrient degradability, and selectively modulate the rumen bacterial community in yaks. Therefore, this study employed a substrate composed of 80% whole-plant corn silage and 20% highland barley straw to systematically investigate the effects of varying isoacid dosages on in vitro rumen fermentation parameters, nutrient degradability, and bacterial community diversity in yaks. The objective of this study was to determine the optimal isoacid supplementation dosage and its regulatory function on rumen fermentation, thereby providing a theoretical basis and technical support for improving roughage utilization efficiency in yak production systems.

## 2. Materials and Methods

### 2.1. Mixed Isoacid Supplement, Substrate Preparation, and Rumen Fluid Collection

The mixed isoacid supplement used in the experiment is a commercially available feed additive product supplied by Sichuan Action Biotech Co., Ltd. (Guanghan, China). It appears as a white powder with a pungent odor, which has an effective content of 64% and consists of 2-methylbutyrate, valerate, isovalerate, and isobutyrate in a mass ratio of 2:2:1:3.

Whole-plant corn silage was collected in Litang County, Sichuan Province (local altitude: 4100 m) in October 2023, and highland barley straw was collected in Xiangcheng County, Sichuan Province (local altitude: 2600 m) in September 2023. Approximately 500 g of each sample were dried in an oven at 65 °C, subsampled using the quartering method, ground to pass through a 40-mesh sieve, and stored in a ventilated dry place at room temperature.

Rumen fluid was collected from five healthy male Maiwa yaks (aged 4–5, body weight approximately 300 kg). The dietary composition and nutritional levels of the donor animals are provided in Table 1. Immediately after slaughter, rumen contents were obtained from the rumen. The fluid was filtered through four layers of gauze and transferred into a pre-warmed thermos flask, and carbon dioxide was flushed to displace oxygen, maintaining an anaerobic environment. The flask was sealed, transported to the laboratory without delay, and used immediately for the in vitro gas production experiment.

### 2.2. In Vitro Fermentation Experiment and Sample Collection

The design of the experiment was a single-factor design. Five isoacid supplementation levels were set, with six replicates per treatment, namely, 0, 0.1%, 0.2%, 0.3%, and 0.4% of the substrate on an air-dry basis. The mixed roughage (80% whole-plant corn silage and 20% highland barley straw on the air-dry basis) used as substrate, and three blank samples were set up for GP correction. The artificial buffer solution was prepared according to the method described by Menke et al. [12]. Rumen fluid was uniformly mixed with the artificial buffer solution at a 1:2 (*v*/*v*) ratio, and carbon dioxide was continuously flushed during the mixing process to maintain an anaerobic environment, thus forming the in vitro fermentation mixture. A quantity of 100 mL of the above mixture was injected into syringes containing the substrate and corresponding doses of isoacids. After expelling residual gas from the syringes, they were sealed and placed in a constant temperature shaker at 39 °C for 72 h of in vitro fermentation, and samples were collected upon completion of in vitro fermentation.

During in vitro fermentation, cumulative GP was recorded at 8, 16, 24, 36, 48, and 72 h, respectively. The syringes were immersed in ice water to terminate the fermentation at the end of in vitro fermentation. The fermentation fluid was filtered through nylon cloth, and the filtrate was divided into 8 × 10 mL centrifuge tubes and stored at −80 °C for subsequent determination of NH_3_-N, MCP, VFA chemical content, and 16S rRNA gene sequencing. The nylon cloth with residues was used to determine the dry matter degradability (DMD), neutral detergent fiber degradability (NDFD), and acid detergent fiber degradability (ADFD).

### 2.3. Chemical Content and Fermentation Parameters Determination

The pH of the fermentation supernatant was immediately measured using a portable pH meter (AZOVTES AE8601, Dongguan Frank Technology Co., Ltd., Dongguan, China) The DM content of the fermentation residue was determined following the AOAC method [13]. The NDF and ADF contents of the fermentation residue were analyzed using a fiber analyzer (automatic fiber determination analyzer, Gernardt F12) in accordance with the Van Soest method [14]. Lastly, the calculation formulas for DMD, NDFD and ADFD are as follows:

DMD (%) = [(DM of sample-DM of residue)/DM of sample] × 100

NDFD (%) = [(NDF of sample-NDF of residue)/NDF of sample] × 100

ADFD (%) = [(ADF of sample-ADF of residue)/ADF of sample] × 100

The content of NH_3_-N in the fermentation fluid was determined using the colorimetric method by Broderick et al. [15]. Briefly, the 5 mL of fermentation fluid was centrifuged at 1200× *g* for 10 min, and 0.4 mL of the supernatant was added with 2 mL phenol chromogenic agent and 2 mL hypochlorite sequentially and shaken well, and the color was developed for 10 min.

A quantity of 2 mL of fermentation fluid was centrifuged at 2400× *g* for 10 min; another 1 mL of the supernatant was mixed with 0.2 mL of 25% metaphosphoric acid, left to stand overnight at 4 °C, then centrifuged at 9600× *g*, 4 °C for 10 min to prepare the sample, and the content of VFAs was determined by gas-liquid chromatography (Agilent 7890B, Palo Alto, CA, USA) following the method described by Zhang et al. [16].

The content of MCP in the fermentation fluid was measured as follows: 5 mL of fermentation fluid was ultrasonicated (350 w, 15 s per cycle, 5 s interval, 9 cycles) and centrifuged at 1200× *g*, 4 °C for 10 min. 1 mL of the supernatant was centrifuged again at 13,800× *g*, 4 °C for 20 min. The precipitate was rinsed twice with 1 mL of saline, then resuspended in 1 mL of distilled water as test solution. The determination was performed according to the instructions of the BCA Kits (Beijing Solarbio Science & Technology Co. Ltd., Beijing, China).

### 2.4. Ruminal Fluid DNA Extraction, Bacterial Amplicon Sequencing, and Bioinformatics Analysis

The total microbial genomic DNA in rumen fluid was extracted using the E.Z.N.A.^®^ Soil DNA Kit (Omega Bio-tek, Norcross, GA, USA) according to the manufacturer’s instructions. After extraction, the concentration, quality, and integrity of the extracted DNA were detected using a micro-spectrophotometer (NanoDrop2000, Thermo Scientific, Waltham, MA, USA) and 1% agarose gel electrophoresis, respectively. The hypervariable region V3-V4 of the bacterial 16S rRNA was amplified by PCR using the extracted DNA as a template, with forward primer 338F (5′-ACTCCTACGGGAGGCAGCAG-3′) and reverse primer 806R (5′-GGACTACHVGGGTWTCTAAT-3′). PCR was performed on a T100 Thermal Cycler (BIO-RAD, Hercules, CA, USA) via the following procedures: initial denaturation at 95 °C for 3 min, followed by 27 cycles of 95 °C denaturation for 30 s, 55 °C annealing for 30 s, and 72 °C extension for 30 s; and a single extension at 72 °C for 10 min, and ending at 4 °C. The PCR products were detected by 2% agarose gel electrophoresis. Qualified PCR products were purified using a PCR Clean-Up Kit (YuHua, Shanghai, China) according to the manufacturer’s instructions. After quantification with a Qubit 4.0 (Thermo Fisher Scientific, Waltham, MA, USA), the purified amplicons were pooled in equimolar amounts. Paired-end sequencing was performed by Majorbio Bio-Pharm Technology Co., Ltd. (Shanghai, China) on an Illumina NextSeq 2000 platform (Illumina, San Diego, CA, USA) following standard protocols. Reads were quality-trimmed at 3′ and 5′ ends using Fastp [17] to remove low-quality sequences and retain high-quality sequences, which were then assembled with MEGAHIT [18]. High-quality sequences were denoised using DADA2 [19] in QIIME 2 [20], and the resulting denoised sequences were referred to amplicon sequence variants (ASVs). To reduce the impact of sequencing depth on bacterial diversity analysis, the number of 16S rRNA gene sequences for each sample was diluted to 50,201, at which point the average Good’s coverage rate was still 99.80%. Data analysis was performed using the Majorbio Cloud Platform (https://www.majorbio.com/, accessed on 27 November 2025) and the Weikemeng Platform (https://www.bioincloud.tech/standalone-task-ui/corheatmap, accessed on 27 November 2025). The Kruskal–Wallis rank-sum test was used to compare the Alpha diversity indices among the three groups of samples, including Sobs, Simpson, Shannon and Chao indices. Based on the Bray–Curtis distance matrix, principal coordinate analysis (PCoA) was conducted, and PCoA plots were generated to assess Beta diversity. For species difference analysis, linear discriminant analysis (LDA) was performed using LEfSe [21], with the threshold of LDA effect size set to greater than 2. Mantel-test analysis was used to explore the correlations between the differential species identified by LDA and rumen fermentation parameters, and a mantel-test network heatmap was generated [22].

### 2.5. Calculations and Statistical Analysis

The kinetics parameters of in vitro gas production was analyzed using the nonlinear model developed by Groot et al. [23], according to the following equation:

GP = A/[1 + (C/t) B]

In this equation, GP is the cumulative gas production (mL/2000 mg) at time “t”; A is the asymptotic gas production (mL/2000 mg), indicating the maximum ability of fermentation; B is a sharpness parameter that defines the shape of curve; C is the time at which half of the asymptotic gas production. The parameters “A”, “B”, and “C” were calculated by non-linear regression using SPSS 26.0 software (version 26.0; IBM Crop., Armonk, NY, USA).

The normality of all data distributions was assessed using the Shapiro–Wilk test. For data that met the normality assumption, parametric analyses were applied. Specifically, cumulative gas production at each time point was analyzed using a two-way repeated-measures ANOVA with SPSS 26.0 software (version 26.0; IBM Crop., USA), with the sphericity assumption checked by Mauchly’s test and the Greenhouse–Geisser correction applied when violated. All other normally distributed data were analyzed using the PROC MIXED procedure in SAS (version 9.4; SAS Institute Inc., Cary, NC, USA), with rumen fluid as a random effect. For data that did not meet the normality assumption (including NH_3_-N, acetate, isobutyrate, and A:P), non-parametric tests were used. Multiple comparisons for parametric analyses were performed using the Tukey–Kramer method. Data are presented as mean ± standard error, with statistical significance determined at *p* < 0.05.

## 3. Results

### 3.1. In Vitro Gas Production Kinetics and Nutrient Degradability

**Figure 1:**
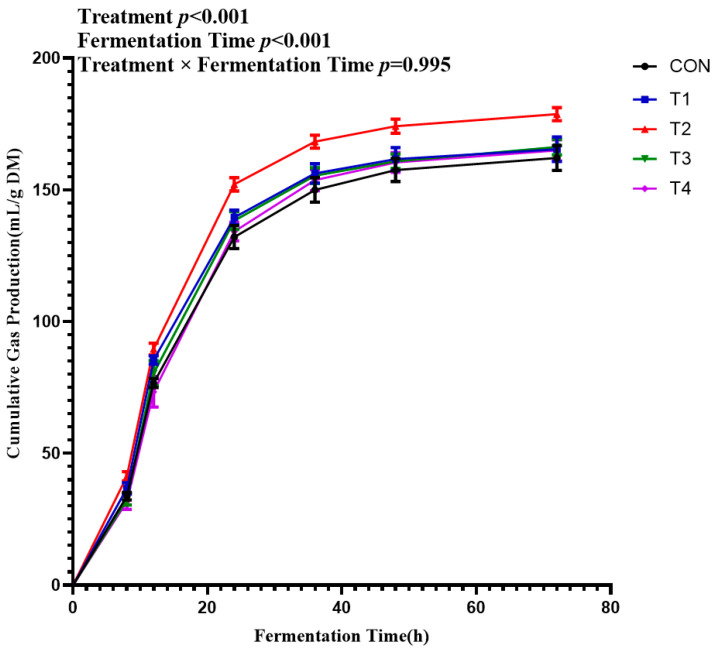
Cumulative gas production of in vitro fermentation experiment. CON was supplemented without isoacids; T1, T2, T3, T4 were supplemented with 0.1%, 0.2%, 0.3%, 0.4% isoacids of the substrate of DM, respectively.

Cumulative GP responses to different doses of isoacids are presented in Figure 1. Cumulative GP increased significantly with fermentation time (*p* < 0.001), reaching the highest value at 72 h, which was significantly higher than other time points (*p* < 0.05). A quadratic trend of first increasing and then decreasing was observed in cumulative GP with increasing isoacid supplementation (*p* < 0.001). Compared with the control, supplementation with isoacids at 0.1% and 0.2% of substrate significantly increased cumulative GP (*p* < 0.05). Furthermore, cumulative GP in the 0.2% isoacids group was significantly higher than that in the 0.1%, 0.3% and 0.4% isoacids groups (*p* < 0.05). No significant interaction was observed between fermentation time and isoacid doses (*p* = 0.995). As shown in Table 2, all GP kinetic parameters responded quadratically to increasing isoacid doses (*p* < 0.05), with the 0.2% isoacids group achieving the highest asymptotic gas production “A”. Table 3 presents the in vitro rumen DMD, NDFD, and ADFD across different isoacid treatments. There were no significant differences in DMD, NDFD, and ADFD among all groups (*p* > 0.05).

### 3.2. In Vitro Fermentation Parameters

The effects of different isoacid supplementation doses on in vitro rumen fermentation parameters are presented in Table 4. The contents of MCP, acetate, propionate, and TVFA all showed a quadratic response to increasing isoacid doses (*p* < 0.05). Compared with the control, supplementation with 0.2% isoacids significantly increased MCP, acetate, and TVFA contents (*p* < 0.05), and tended to increase propionate content (0.05 < *p* < 0.1). However, no significant differences were observed among groups in pH, NH_3_-N content, butyrate content, valerate content, isobutyrate content, isovalerate content, or the acetate-to-propionate ratio (*p* > 0.05).

### 3.3. Bacterial Community Diversity and Composition Analysis

A total of 30,097 ASVs and 948,786 high-quality reads were obtained from 18 samples (CON, T2 and T4 groups), with an average of 52,710 reads per sample. After denoising, the number of reads in each sample was rarefied to the minimum sequences count of 31,518, resulting in 19,707 ASVs.

**Figure 2:**
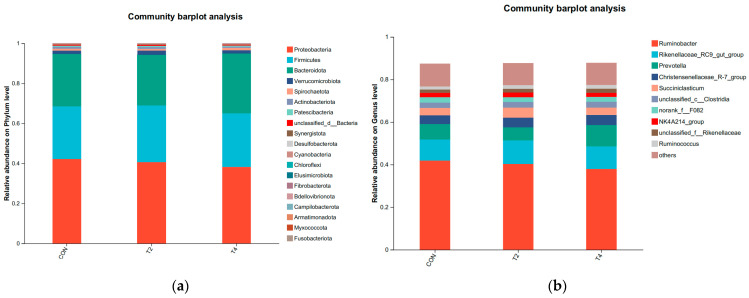
Relative abundance of ruminal microbiota at the (**a**) phylum and (**b**) genus levels. CON was supplemented without isoacids; T2 was supplemented with 0.2% isoacids of the substrate of DM; T4 was supplemented with 0.4% isoacids of the substrate of DM.

The composition of ruminal bacterial communities at the phylum and genus levels is shown in Figure 2. No significant differences in bacterial community composition were found among the three groups at either the phylum or genus level (*p* > 0.05). At the phylum level, *Proteobacteria*, *Firmicutes*, and *Bacteroidota* were the dominant phyla across all groups. At the genus levels, the dominant genera included *Ruminobacter*, *Rikenellaceae*_RC9_gut_group, *Prevotella*, *Christensenellaceae*_R-7_group, and *Succiniclasticum*.

**Figure 3:**
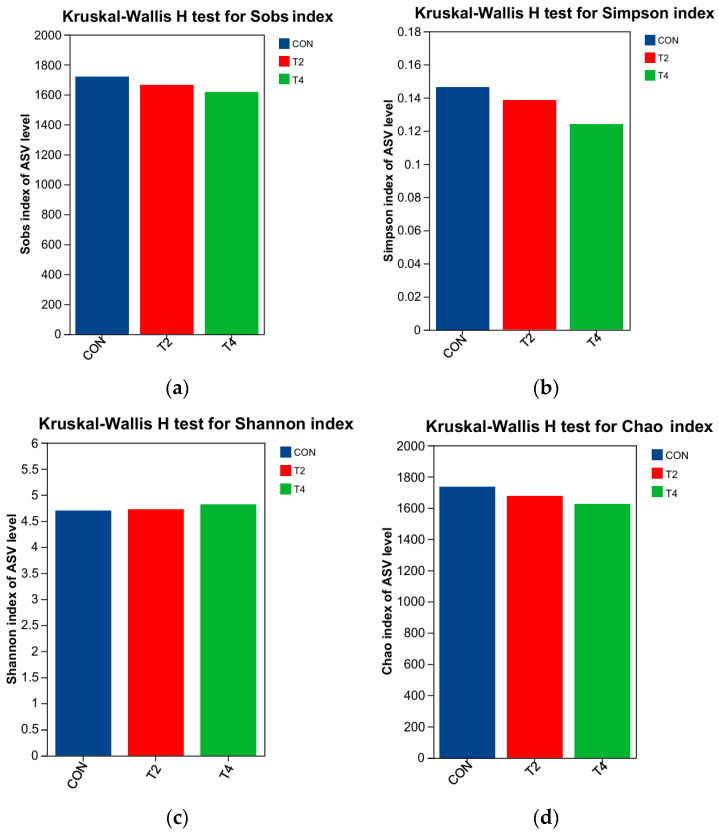
Effects of supplementation with different isoacid dosages on the alpha diversity of rumen microbiota in the rumen fluid of yaks. (**a**) Sobs index; (**b**) Simpson index; (**c**) Shannon index; (**d**) Chao index. CON was supplemented without isoacids; T2 was supplemented with 0.2% isoacids of the substrate of DM; T4 was supplemented with 0.4% isoacids of the substrate of DM.

**Figure 4:**
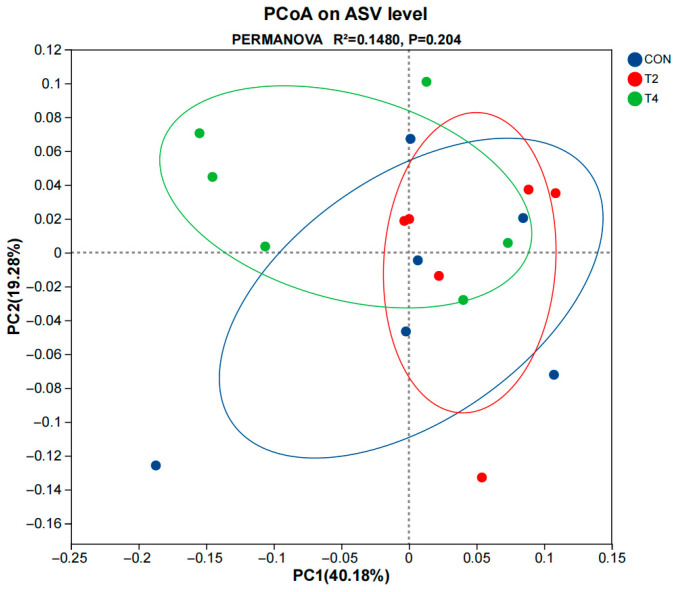
Principal coordinate analysis (PCoA) of rumen microbiota based on the Bray–Curtis distance metric. CON was supplemented without isoacids; T2 was supplemented with 0.2% isoacids of the substrate of DM; T4 was supplemented with 0.4% isoacids of the substrate of DM.

Alpha diversity analysis (Figure 3) was used to evaluate bacterial community richness (Chao and Sobs indices) and diversity (Shannon and Simpson indices) among the three groups. No significant differences in these indices were observed between the CON, T2, and T4 groups (*p* > 0.05). PCoA based on the Bray–Curtis distance (Figure 4) also indicated no significant differences in beta diversity among the CON, T2, and T4 groups (PERMANOVA, *R*^2^ = 0.148, *p* = 0.204).

**Figure 5:**
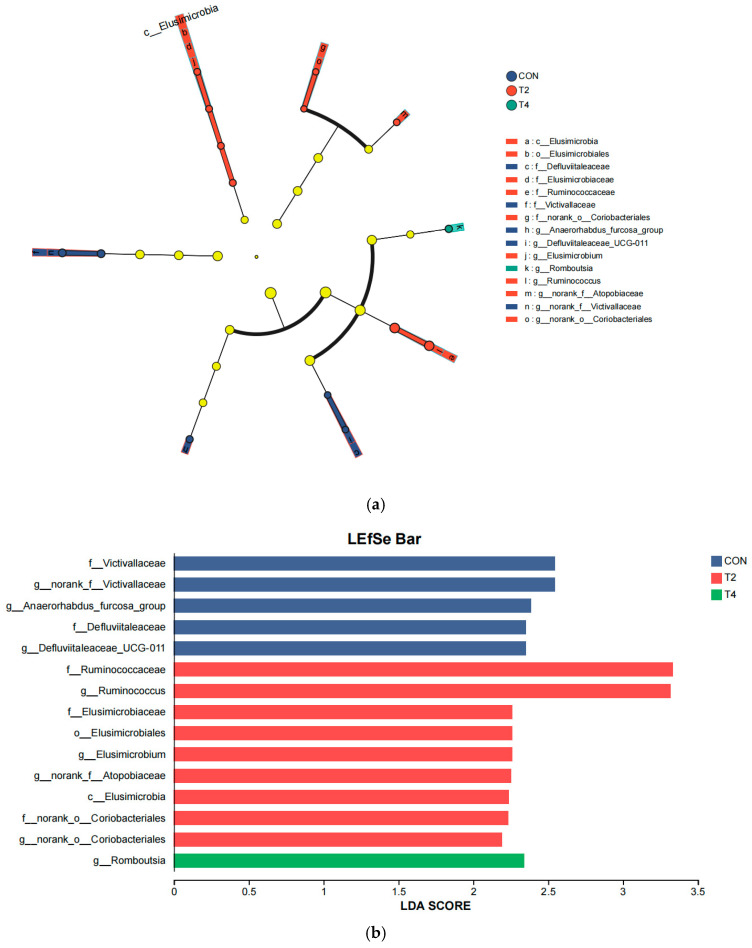
Effects of isoacid supplementation on the discriminative rumen microbiota. (**a**) Cladogram of the three groups from phylum to genus level; (**b**) Linear discriminant analysis of rumen microbiota effects. CON was supplemented without isoacids; T2 was supplemented with 0.2% isoacids of the substrate of DM; T4 was supplemented with 0.4% isoacids of the substrate of DM.

LEfSe was performed to identify bacterial biomarker taxa responses to different isoacid doses (Figure 5). In the control group, f_*Victivallaceae*, g_norank_f_*Victivallaceae*, f_*Defluviitaleaceae*, g_*Defluviitaleaceae*_UCG-011, and g_*Anaerorhabdus_furcosa*_group were significantly enriched (*p* < 0.05). In the T2 group, f_*Ruminococcaceae*, g_*Ruminococcus*, f__*Elusimicrobiaceae*, o__*Elusimicrobiales*, g__*Elusimicrobium*, g_norank_f_*Atopobiaceae*, c_*Elusimicrobia*, g_norank_o_*Coriobacteriales*, and f_norank_o_*Coriobacteriales* showed significantly enriched (*p* < 0.05). In the T4 group, g_*Romboutsia* was significantly enriched (*p* < 0.05).

**Figure 6:**
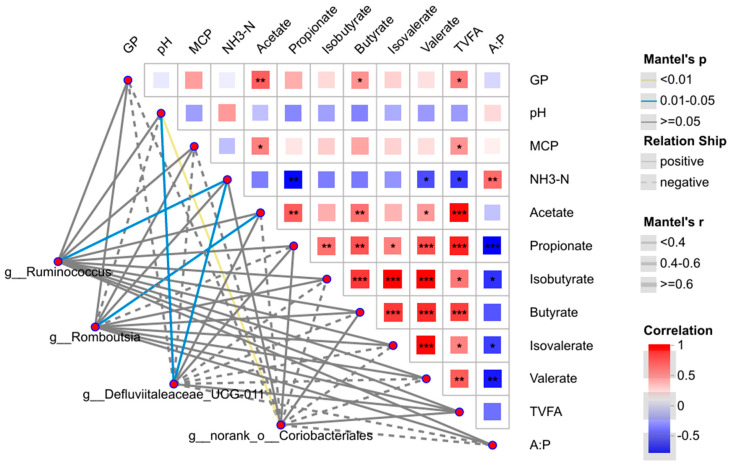
Mantel-test network heatmap between the differential species identified by LDA at the genus level with rumen fermentation parameters. * *p* < 0.05, ** *p* < 0.01, *** *p* < 0.001.

Mantel-test analysis was conducted to explore correlations between the differential bacterial genera identified by LDA and rumen fermentation parameters (Figure 6). Four differential bacterial genera showed significant correlations with rumen fermentation parameters: g_*Ruminococcus* abundance was significantly positively correlated with NH_3_-N content (*r* < 0.4, *p* < 0.05); g_*Romboutsia* abundance was significantly positively correlated with acetate content (*r* < 0.40, *p* < 0.05); g_*Defluviitaleaceae*_UCG-011 abundance was significantly positively correlated with both NH_3_-N content and rumen pH (*r* < 0.40, *p* < 0.05); g_norank_o_*Coriobacteriales* abundance was highly significantly positively correlated with rumen pH (*r* < 0.40, *p* < 0.01).

## 4. Discussion

GP serves as a key indicator of the rumen microbial fermentation extent and feed degradability [24,25]. In the present study, cumulative GP showed a significant quadratic response to increasing isoacid doses, peaking at 0.2% isoacid supplementation. Isoacids could stimulate the growth and activity of both fibrolytic and non-fibrolytic bacteria [26,27], thereby promoting the degradation of carbohydrates and enhancing the production of fermentation gases. No significant differences in nutrient degradation rates were observed among all groups in this study. Therefore, it is speculated that 0.2% supplementation dosage of isoacids activates non-fibrolytic bacteria, accelerates the degradation of non-fibrous carbohydrates, and consequently leads to a significant increase in GP. This suggests an optimal isoacid supplementation dosage for enhancing ruminal fermentation, beyond which additional supplementation provides no further benefit or may become inhibitory, potentially due to the inhibitory effects of high isoacid concentrations on microbial activity or community balance, indicating that the effect of isoacid supplementation is dose-dependent. This dose-dependent enhancement of rumen fermentation capacity is further supported by the GP kinetic, where 0.2% isoacid supplementation achieved the highest asymptotic gas production “A”. However, findings across studies are inconsistent; for example, Jiang et al. [11] reported that isoacid supplementation did not affect in vitro cumulative GP when using a TMR for yaks with a concentrate-to-forage ratio of 50:50 as substrate. This discrepancy suggests that the effect of isoacids on in vitro rumen GP may be influenced by the concentrate-to-forage ratio of the substrate. The optimal dosage not only enhanced GP but also positively influenced other key parameters of rumen fermentation, as detailed below.

Rumen pH is a key fermentation parameter closely associated with rumen function [28]. In this study, pH remained within the normal range (typically 5.3–6.8) across all groups [29,30], indicating that the isoacid supplementation did not adversely affect the rumen fermentation environment of yaks. This stability is notable, given that rumen pH is susceptible to fluctuations induced by factors such as dietary concentrate-to-forage ratio and exogenous additives [31,32]. For instance, Wang et al. [33] reported that supplementing 2-methylbutyrate significantly decreased rumen pH in Chinese Simmental steers. This discrepancy suggests that the effect of isoacids on rumen pH may vary with animal species and the composition of isoacids.

The utilization of dietary protein is reflected in ruminal NH_3_-N and MCP levels [34,35]. No significant differences in NH_3_-N content were observed among groups, which might be related to the low protein content of the substrate. However, MCP content was significantly higher in the 0.2% isoacids group than in the control group. This may be attributed to the fact that isoacids act as ruminal metabolites of branched-chain amino acids, which can be utilized by ruminal fibrolytic bacteria to promote microbial cell proliferation and enzyme activity, enhance fiber degradation, and improve carbon source availability in the rumen, thereby increasing microbial protein synthesis [36,37,38]. Cummins et al. [39] added isoacids to a substrate with a crude protein level of 13% and significantly increased MCP content, while Mitchell et al. [9] found that supplementing 2.15 mmol/d of isoacids in a high-roughage diet can significantly improve MCP synthesis efficiency. These studies indicated that the effect of isoacids on rumen MCP synthesis may be influenced by factors such as isoacid supplementation dose and the crude protein or crude fiber level of the substrate. Concurrently, the energy metabolism in the rumen, indicated by VFA production, was also enhanced at this optimal dosage.

VFAs provide 70–80% of the energy required for ruminant growth and production [40], and their concentration and composition are key indicators of rumen fermentation capacity and status [41,42]. The present study showed that 0.2% isoacid supplementation significantly increased the TVFA and acetate contents, along with a tendency to increase propionate. This aligns with previous reports that isoacid supplementation can promote rumen VFAs production. For example, Hemsley et al. [43] found that supplementation with 0.56% mixed isoacids increased TVFA content in the rumen of Merino rams. Wang et al. [44] reported that supplementation with 2-methylbutyrate in the diet of Simmental steers significantly increased TVFA content and the molar proportion of butyrate in the rumen. Collectively, the improvements in GP, MCP, and VFAs at the 0.2% isoacid supplementation dosage were associated with concurrent changes in the rumen bacterial community.

The improved fermentation parameters at the optimal 0.2% isoacid supplementation corresponded with significant changes in the rumen bacterial community. LEfSe analysis revealed that the T2 group was significantly enriched with bacterial biomarkers such as g_*Ruminococcus*, g__*Elusimicrobium*, g_norank_f_*Atopobiaceae*, and g_norank_o_*Coriobacteriales*. *Ruminococcus* is a core fiber-degrading genus in the rumen that secretes enzymes to degrade cellulose and hemicellulose for VFAs synthesis [45]. *Elusimicrobium* contains key enzyme genes involved in core carbohydrate metabolism pathways and participates in VFA production by catalyzing carbohydrate metabolism [46]. *Coriobacteriales* is associated with the production of short-chain fatty acids (SCFAs) [47,48]. *Atopobiaceae* is a beneficial bacterium, which associated with the production of SCFAs [49]. Overall, the T2 group was significantly enriched with acidogenic bacteria, which provides a plausible microbial mechanism for the enhanced TVFA production observed at this dosage. The result suggested that 0.2% isoacid supplementation may promote VFA synthesis by increasing the abundance of acidogenic bacteria.

Mantel-test analysis revealed a significant positive correlation between g_*Ruminococcus* abundance and NH_3_-N content. Since the growth of *Ruminococcus* is dependent on the nitrogen source supply [50,51], this correlation may suggest that the nitrogen-rich fermentation environment fostered by 0.2% isoacid supplementation is favorable for its proliferation. Additionally, a highly significant positive correlation was observed between g_norank_o_*Coriobacteriales* abundance and rumen pH. Given that *Coriobacteriales* is known to be associated with SCFA production [47,48], this correlation could be related to its role in SCFA metabolism, which influences ruminal pH.

## 5. Conclusions

Supplementation with 0.2% isoacids improved in vitro rumen fermentation and roughage utilization in yaks using a substrate based on corn silage–highland barley straw, as evidenced by increased gas production, microbial protein, acetate, and total volatile fatty acids. These beneficial effects were associated with modulation of key bacterial biomarker taxa, including g_*Ruminococcus*, g_*Elusimicrobium*, g_norank_f_*Atopobiaceae*, *and* g_norank_o_*Coriobacteriales*. These findings warrant further validation via in vivo trials and may represent a promising nutritional strategy to support sustainable yak production on the Qinghai–Tibet Plateau.

## Figures and Tables

**Table 1:** Composition and nutritional level of diets (DM basis).

Dietary Compositions	% of DM	Nutrient Levels ^(1)^	% of DM
Corn	31.0	DM ^(3)^	93.3
Soybean meal	13.0	EE	3.15
Urea	0.15	CP	14.3
Na_2_SO_4_	0.35	NDF	30.5
NaCl	0.35	ADF	12.1
Premix ^(2)^	1.90	Ca	0.76
Rumen—protected lysine	0.15	P	0.38
Rumen—protected methionine	0.10		
Yeast culture	0.50		
Small peptide	0.50		
Hydrogenated palm oil	2.00		
Corn silage	50.0		
Total	100.0		

Note: ^(1)^ DM, dry matter; EE, ether extract; CP, crude protein; NDF, neutral detergent fiber; ADF, acid detergent fiber; Ca, calcium; P, phosphorus. ^(2)^ The premix provided the following per kg of the diet: vitamin A, 2500 IU; vitamin D, 550 IU; vitamin E, 10 IU; Cu, 10 mg; Fe, 50 mg; Mn, 40 mg; Zn, 40 mg; I, 0.5 mg; Se, 0.2 mg; Co, 0.2 mg. Dietary compositions were adopted from a previous study [11]. Nutrient levels were measured values. ^(3)^ The unit for DM is %.

**Table 2:** Effects of isoacid supplementation to roughage on the in vitro kinetics parameters.

Items ^(1)^	Groups ^(2)^					SEM	*p*-Value ^(3)^		
	CON	0.10%	0.20%	0.30%	0.40%		T	L	Q
A (mL/2000 mg AD)	323.6	329.8	356.0	328.5	330.4	3.945	0.065	0.631	0.048
B	2.67	2.83	2.80	2.95	2.73	0.034	0.086	0.264	0.037
C (h)	13.0	12.3	12.2	12.7	13.4	0.170	0.139	0.251	0.020

Note: ^(1)^ A, the asymptotic gas production (mL/2000 mg AD); B, the sharpness parameter; C, the time at which half of the asymptotic gas is produced (h). ^(2)^ CON was supplemented without isoacids; 0.10%, 0.2%, 0.3%, 0.4% was supplemented with 0.1%, 0.2%, 0.3%, 0.4% isoacids of the substrate of DM, respectively. ^(3)^ T, treatment; L, linear; Q, quadratic.

**Table 3:** Effects of isoacid supplementation to roughage on nutrient degradation rates in vitro.

Items ^(1)^	Groups ^(2)^					SEM	*p*-Value ^(3)^		
	CON	0.1%	0.2%	0.3%	0.4%		T	L	Q
DMD	49.7	50.7	51.1	50.3	49.6	1.602	0.909	0.907	0.728
NDFD	47.4	49.2	48.7	48.3	48.7	1.731	0.981	0.729	0.866
ADFD	26.8	33.0	32.0	31.6	30.4	2.526	0.487	0.477	0.254

Note: ^(1)^ DMD, dry matter degradability; NDFD, neutral detergent fiber degradability; ADFD, neutral detergent fiber degradability. ^(2)^ CON was supplemented without isoacids; 0.10%, 0.2%, 0.3%, 0.4% was supplemented with 0.1%, 0.2%, 0.3%, 0.4% isoacids of the substrate of DM, respectively. ^(3)^ T, treatment; L, linear; Q, quadratic.

**Table 4:** Effect of isoacid supplementation to roughage on rumen fermentation parameters in vitro.

Items ^(1)^	Groups ^(2)^					SEM	*p*-Value ^(3)^		
	CON	0.10%	0.20%	0.30%	0.40%		T	L	Q
pH	5.56	5.55	5.56	5.54	5.58	0.012	0.122	0.339	0.172
MCP (mg/mL)	0.58 ^b^	0.65 ^ab^	0.76 ^a^	0.64 ^b^	0.66 ^ab^	0.037	0.027	0.272	0.046
NH_3_-N (mg/dL)	26.2	24.7	23.9	24.2	25.9	1.333	0.673	0.804	0.319
VFAs (mmoL/L)									
Acetate	75.1 ^b^	81.5 ^ab^	88.6 ^a^	79.4 ^ab^	74.9 ^b^	3.12	0.040	0.830	0.011
Propionate	29.7	33.1	34.7	30.0	28.8	1.634	0.080	0.406	0.040
Butyrate	13.0	12.1	14.5	11.8	11.2	0.876	0.114	0.198	0.198
Valerate	2.53	2.44	2.75	2.4	2.38	0.185	0.618	0.556	0.649
Isobutyrate	2.22	2.03	2.31	2.06	2.07	0.145	0.654	0.844	0.829
Isovalerate	3.69	3.42	3.98	3.33	3.37	0.308	0.706	0.457	0.667
TVFA	126.2 ^b^	134.6 ^ab^	146.9 ^a^	129.1 ^b^	122.8 ^b^	5.450	0.038	0.537	0.023
A:P	2.56	2.48	2.58	2.64	2.62	0.099	0.752	0.360	0.639

Note: ^a,b^ Different letters in the same row indicate a significant difference between treatments (*p* < 0.05). ^(1)^ MCP, microbial protein; NH_3_-N, ammonia nitrogen; VFAs, volatile fatty acids; TVFA, total volatile fatty acids; A:P, acetate: propionate ratio; SEM, standard error of the mean. ^(2)^ CON was supplemented without isoacids; 0.10%, 0.2%, 0.3%, 0.4% was supplemented with 0.1%, 0.2%, 0.3%, 0.4% isoacids of the substrate of DM, respectively. ^(3)^ T, treatment; L, linear; Q, quadratic.

## Data Availability

16s rRNA sequencing raw data were deposited in the Genome Sequence Archive (GSA), under accession number CRA035635 that can be found at https://ngdc.cncb.ac.cn/gsa/ (accessed on 22 December 2025).
